# Can supplementary private health insurance further supplement health

**DOI:** 10.3389/fpubh.2022.961019

**Published:** 2022-09-27

**Authors:** Xinlin Chen, Dandan Guo, Huawei Tan, Yunfan Zhang, Yanchen Liu, Xinlan Chen, Yingchun Chen

**Affiliations:** ^1^School of Medicine and Health Management, Tongji Medical College, Huazhong University of Science and Technology, Wuhan, China; ^2^Research Centre for Rural Health Service, Key Research Institute of Humanities and Social Sciences of Provincial Department of Education, Wuhan, China

**Keywords:** private health insurance, social health insurance, health promotion, EQ-5D, instrumental variable

## Abstract

**Background:**

China advocates a health insurance system with social health insurance (SHI) as the main body and private health insurance (PHI) as the supplement. The study of PHI's complementary role in health is conducive to providing evidence for PHI's policy expansion and encouraging the public to participate in PHI, which is insufficient in China.

**Methods:**

We used the three-wave balanced panel data of the China Health and Retirement Longitudinal Survey (CHARLS). Taking the ownership of supplementary PHI as the independent variable and EQ-5D index scores as the dependent variable, the panel instrumental variable (IV) method was used to analyze the impact of participation in PHI on health. We also assessed the heterogeneity of the health effects of PHI between chronic and non-chronic disease groups and between low- and high-income groups.

**Results:**

The coverage rate of PHI at baseline was 10.53%. The regression results showed that participating in PHI on the basis of SHI could result in an additional 8.21% health gain (*p* < 0.001). At the same time, PHI had greater health gain for chronic disease population than for healthy population (9.25 vs. 6.24%, *p* < 0.001), and greater health gain for high-income population than for low-income population (8.32 vs. 5.31%, *p* < 0.001).

**Conclusion:**

Participating in supplementary PHI can effectively enhance the health status of the insured, and has a more significant effect on patients with chronic diseases. The development of PHI should be further supported, while the health inequality in different income groups should be paid attention to.

## Introduction

As a financial sharing mechanism of medical expenses, medical insurance can alleviate the economic losses caused by medical risks and make those who do not have the ability to seek medical treatment get timely services. Therefore, a good insurance system is crucial to obtain high-quality medical services and improve health (1). China has developed the world's largest medical security system, with government-led SHI covering 1.36 billion people, accounting for over 95% of the country's population (2, 3). However, due to the limited pool of SHI funds, the National Healthcare Security Administration haslimited the scope of SHI reimbursement for drugs, treatment items and medical service facilities, and formulated the deductibles, copayments, and maximum allowable costs (4, 5), making it difficult for SHI to meet the growing health needs of the population and limiting the ability of SHI to withstand catastrophic health expenditure. In 2021, the personal health expenditure accounted for 27.7% of the total health expenditure in China, much higher than the proportion of poverty control expenditure proposed by the World Health Organization of 15–20% (6). In this context, the Chinese government clearly proposes to take PHI as a supplement to SHI. PHI adopts the voluntary principle and relies on contracts to cover expenses and high-quality services beyond the reimbursement scope of SHI. The mainstream PHI products in China are currently characterized by reimbursing expensive services such as hospitalization for critical illness that are not part of SHI benefits. It can effectively compensate for the shortage of SHI, reduce the high medical expenses of individuals, enhance residents' ability to invest in health, and guarantee residents' higher health needs (7, 8).

Although the Chinese government is paying more attention to the role of PHI as a source of funding for the health system and giving policy support (4), the current coverage rate of PHI in China remains extremely low, only roughly 10%, whereas the proportion of PHI in in developed countries are range from 20 to 30% (9). As a supplementary role to the national health insurance system, PHI plays a limited supporting role at present, and the huge market space for PHI in China requires further development (10). As the development of PHI in China remains in its infancy, only few studies have systematically evaluated the impact of PHI on the health of insured Chinese residents. The existing studies on the role of PHI on health are controversial. While some studies have demonstrated the effect of PHI in promoting health by guaranteeing health service utilization (11, 12), others disagree. They claim that the causality may be an illusion because factors such as adverse selection by purchasers and cream skimming by insurers may lead to reverse causality of the insured's health status on PHI participation behavior, i.e., there is an endogeneity problem that is not addressed (13, 14). In addition, the effects of insurance on health service utilization and health expenditure are confirmed to vary among different populations, such as across age (15, 16), gender (17, 18), income (19, 20), or ethnic subgroups (21), between sick and healthy people (22), and across geographical locations (23–25), which is less considered in the studies of PHI's effects on health.

Therefore, we try to solve the endogeneity problem by using the panel IV method, estimate the health effect of PHI on the insured, and test the heterogeneity of this effect among different populations, aiming to supplement the real-world evidence for the effect of PHI and provide the basis for the improvement of PHI policy. Based on the previous studies, we hypothesize that: ①compared with residents with only SHI, residents with supplementary PHI can further improve their health level; ② PHI provides greater health gains for people with chronic diseases than for people without chronic disease; and ③ PHI provides greater health gains for high-income people than for low-income people.

## Materials and methods

### Data source

The data used in this study were obtained from the CHARLS database (http://charls.ccer.edu.cn/zh-CN), which attempts to create a high-quality public micro-database that can provide a wide range of information on Chinese residents, from socioeconomic status to health status. The national baseline survey was conducted in 2011–2012, with wave 2 in 2013, wave 3 in 2015, and wave 4 in 2018.

Data from waves 2, 3, and 4 were selected for analysis in this study, and balanced panel data were formed based on the following criteria: (1) the sample was fully traceable in all three periods and (2) no explanatory variables or key explanatory variables were missing. Samples that did not meet the criteria were excluded, resulting in 14,799 data for each period, for a total of 44,397 data.

### Study variables

#### Dependent variables

All questions in the CHARLS database were self-reported by respondents. To minimize individual reporting errors, we planned to combine various self-reported conditions into a comprehensive health index and finally decided to use the three-level EuroQol 5-dimension (EQ-5D) index scores as the dependent variable (26, 27). EQ-5D is a standardized instrument for measuring health states, and it is widely used internationally because of its simplicity, ease of use, and high reliability. It consists of five dimensions (i.e., mobility, self-care, usual activities, pain/discomfort, and anxiety/depression) and three levels for each dimension (i.e., 1 = no problems, 2 = some/moderate problems, and 3 = extreme problems). The five dimensions and three levels can describe 243 unique health states after permutation and combination (28). Liu Guo-en et al. (29) firstly constructed the utility value integral system of EQ-5D based on Chinese population preference by using the time trade-off method. The formula for calculating EQ-5D index scores in the system is as follows.


(1) $$\begin{array}{l} U=1-(0.039+0.099^{*}MO2+0.105^{*}SC2+0.074^{*}UA2\\ +0.092^{*}PD2+0.086^{*}AD2+0.246^{*}MO3+0.208^{*}SC3\\ +0.193^{*}UA3+0.236^{*}P+0.205^{*}AD3+0.022^{*}N3 \end{array}$$

According to Table 1, the score of all 243 health states can be calculated. If all the five dimensions are at level 1, it is 11,111, indicating no health problem and a score of 1. If all the five dimensions are at level 3, it is 33,333, indicating that health is in the worst state and a score of [−0.149, 1].

**Table 1:** The utility value integral system of EQ-5D scale based Chinese residents.

**Variables**	**Definition**	**Coefficient**
*C*	Constant term	0.039
**Mobility**		
MO2	Dimension at level 2	0.099
MO3	Dimension at level 3	0.246
**Self-care**		
SC2	Dimension at level 2	0.105
SC3	Dimension at level 3	0.208
**Usual activities**		
UA2	Dimension at level 2	0.074
UA3	Dimension at level 3	0.193
**Pain/discomfort**		
PD2	Dimension at level 2	0.092
PD3	Dimension at level 3	0.236
**Anxiety/depression**		
AD2	Dimension at level 2	0.086
AD3	Dimension at level 3	0.205
N3	At least one dimension is at level 3	0.022

#### Key variables

The core explanatory variable was the ownership of PHI. It was a binary variable, which was assigned a value of 0 to the sample that only participated in SHI, and 1 to the sample that purchased supplementary PHI on the basis of SHI.

#### Control variables

Based on the existing research results, we selected control variables including socioeconomic characteristics, health behavior and disease status. The definition and codes of variables were shown in the Supplementary File.

### Statistical analysis

We mainly used panel regression method for analysis, and the model is as follows.


(2) $$\begin{array}{l} Y_{it}=\alpha_{0}+\alpha_{1}^{*}X_{it}\;+\alpha_{2}^{*}Z_{it}+u_{i}\;+\varepsilon_{it} \end{array}$$

*Y*_*it*_ represents the EQ-5D index scores of the subject, the core explanatory variable *X*_*it*_ represents whether individual *I* purchased PHI in year *T*, and *Z*_*it*_ represents a series of individual characteristic variables and health behavior variables (In the model 3 and model 4 is the same). The perturbation term is composed of (*u*_*i*_+ ε_*it*_), where *u*_*i*_ is an unobtainable random variable to measure individual heterogeneity, and ε_*it*_ is the error term that varies with individual and time. α_1_ is the core parameter we focused on, indicating the extent of PHI's effect on individual health.

PHI is voluntary, so individuals may decide whether to join PHI by taking into account their income, personality preference, physical conditions, social and cultural environment, and other factors. The omission of these unobtainable variables can lead to the correlation between PHI and disturbance terms. At the same time, “adverse selection” exists in the insurance market. People with high health risks may be more inclined to buy PHI, resulting in two-way causality between explanatory variables and explained variables, which leads to biased results estimation. Therefore, we tried to find an appropriate IV to correct bidirectional causality due to the omission of individual heterogeneity over time and bidirectional causality. Two criteria should be followed when selecting the IV. First, the IV is related to endogenous explanatory variables. Second, the IV is exogenous and unrelated to the disturbance term. A large number of studies identify regional variables related to public policy changes as effective IV (30). Therefore, we chose the average participation rate of PHI in the community as the IV of this study. It may be highly correlated with whether the respondents participate in PHI through the advertising efforts of insurance companies and the externalities of consumption. The insurance coverage in the region has nothing to do with the unobservable information of individuals at the micro level, so it can only affect individual health by influencing the purchase decisions of PHI (31). On this basis, we modified model 2 and constructed a two-stage least-square regression model of the IV and further tested and reported the effectiveness of the IV in subsequent studies.


(3) $$\begin{array}{l} X_{it}=\pi_{1}^{*}Z_{it}\;+\pi_{2}^{*}C_{it}+u_{i}\;+\varepsilon_{it} \end{array}$$

(4) $$\begin{array}{l} Y_{it}=\alpha_{0}+\alpha_{1}^{*}{\hat{X}}_{it}\;+\alpha_{2}^{*}Z_{it}+u_{i}\;+\varepsilon_{it} \end{array}$$

*C*_*it*_ represents the IV, *Z*_*it*_ represents the control variable, and _*it*_ represents the predicted value of *X*_*it*_ obtained from regression of model 3 in the first stage.

To verify the robustness of the results, we replaced the explained variables and used the self-assessed health status of residents in the questionnaire to replace EQ-5D index scores to measure the health level of residents. This variable was measured in the questionnaire with the question “How do you think your health is?”, which was answered using a 5-point Likert scale. We assigned a score of 1, 2, 3, 4, and 5 to “very bad,” “not good,” “fair,” “good,” and “very good,” respectively, and the better the self- assessed health, the higher the score.

All data compilation and statistical analysis were performed using STATA 15.0.

## Results

### Basic characteristics of participants

A total of 14,799 participants in the baseline data in 2013 were all covered by SHI, of which 1,558 were also covered by PHI, accounting for 10.53%. The mean value of EQ-5D index scores for the group with PHI was 0.8289, and that for the group without PHI was 0.8151, with a significant difference in health status between the two groups (*p* < 0.001). Those with supplemental PHI were more likely to be rural (*p* < 0.05) and have a relatively higher per capita household income (*p* < 0.001) (Table 2).

**Table 2:** Sample characteristics of the selected respondents at baseline.

**Variables**	**With PHI** **1,558 (10.53)**	**Without PHI** **13,241 (89.47)**	**Total**	***p***
EQ-5D index scores	0.8289 (0.191)	0.8151 (0.2037)	0.8166 (0.2025)	<0.001^a^
Gender = 1	756 (48.52)	6,250 (47.20)	7,006 (47.34)	0.323^b^
**Age**				0.832^b^
18–44 years	127 (8.15)	1,000 (7.55)	1,127 (7.62)	
45–59 years	745 (47.82)	6,363 (48.06)	7,108 (48.03)	
60–74 years	548 (35.17)	4,733 (35.75)	5,281 (35.68)	
≥75 years	138 (8.86)	1,145 (8.65)	1,283 (8.67)	
Marital Status = 1	1,353 (86.84)	11,514 (86.96)	12,867 (86.95)	0.899^b^
**Education**				0.043^b^
Junior high school and below	1,399 (89.79)	11,640 (87.91)	13,039 (88.11)	
Vocational/technical secondary school/high school	137 (8.79)	1,311 (9.90)	1,448 (9.78)	
Junior college and above	22 (1.41)	290 (2.19)	312 (2.11)	
Non-rural household registration	1,168 (74.97)	10,270 (77.56)	11,438 (77.29)	0.021^b^
Smoke = 1	547 (35.11)	4,557 (34.42)	5,104 (34.49)	0.586^b^
Drink = 1	545 (34.98)	4,486 (33.88)	5,031 (34.00)	0.385^b^
Social contact = 1	958 (61.49)	8,076 (60.99)	9,034 (61.04)	0.704^b^
Chronic disease = 1	985 (63.22)	8,076 (60.99)	9,460 (63.92)	0.542^b^
Household incomes per capita	8.6476 (2.0577)	8.4639 (1.9319)	8.4832 (1.9463)	<0.001^a^

The descriptive results of “EQ-5D index scores” and “Household incomes per capita” were represented by mean (standard deviation), while the descriptive results of other indexes were represented by quantity (proportion).

a Outcomes of Student-*t*-test.

b Outcomes of Chi-square test.

### Analysis of the effects of PHI on health status by panel regression model

Columns 1, 2, and 3 of Table 3 respectively reported the estimation results of the panel pooled model, fixed-effect (FE) model, and random effect (RE) model. The *p-*values of the *F* test of FE and RE were both < 0.001, which strongly rejected the null hypothesis that “pooled regression is acceptable”. That is, individual effect existed, and FE and RE were significantly better than pooled regression. The results of Hausman's test were significant at the 1% significance level (*p* < 0.001), which rejected the null hypothesis that explanatory variables and residuals were not correlated. It was more appropriate to adopt the FE model rather than the RE model. Therefore, we took the regression results of FE model as the analysis standard and the regression results of other two methods as the reference.

**Table 3:** Panel regression results of the effect of having PHI on the health state of residents.

**Variables**	**Pooled model (1)**	**FE model (2)**	**RE model (3)**
The ownership of PHI	0.0101 ^***^ (0.0031)	0.0116 ^***^ (0.0040)	0.0101 ^***^ (0.0031)
Gender	−0.0018 (0.0017)	—	−0.0018(0.0017)
Age	−0.0063^***^ (0.0012)	−0.0403^***^ (0.0034)	−0.0063^***^ (0.0012)
Marital status	0.0018 (0.0025)	0.0039 (0.0030)	0.0018 (0.0025)
Education	0.0033 (0.0022)	0.0024 (0.0035)	0.0033 (0.0022)
Registered permanent residence	−0.0078^***^ (0.0021)	−0.0121^***^ (0.0032)	−0.0078^***^ (0.0021)
Smoke	−0.0058^***^ (0.0018)	−0.0319^***^ (0.0054)	−0.0058^***^ (0.0018)
Drink	0.0424^***^ (0.0017)	0.0434^***^ (0.0022)	0.0424^***^ (0.0018)
Social contact	0.0436 ^***^ (0.0018)	0.0451^***^ (0.0021)	0.0436^***^ (0.0017)
Disease	−0.0622^***^ (0.0017)	−0.0603 ^***^ (0.0021)	−0.0621^***^ (0.0017)
Household income per capita	0.0010 ^***^ (0.0003)	0.0008^**^ (0.0003)	0.0010^***^ (0.0003)
*F* test: *F*	0.0000^*^	195.09^***^	38.68^***^
Hausman test: *χ^2^*		194.50^***^
Observations		44,397

Regression results were expressed by coefficients (standard error).

*** ,

** and

* represented significant at 1%, 5% and 10% levels respectively.

“—”, The binary variable “Gender” was automatically missing in the panel regression.

The following analysis results were presented in the same way.

The regression results of the FE model in column 2 revealed a significant positive correlation between the purchase of supplementary PHI and the health level of residents. The results of the three methods showed an effect coefficient of approximately 0.01, indicating that participating in PHI on top of SHI could bring a health utility return of 1% (Table 3).

### Analysis of the effects of PHI with excluding endogenous

The use of IV presupposes the existence of endogenous explanatory variables, for which a Hausman test was conducted. The results showed that the original hypothesis of “all explanatory variables are exogenous” could be rejected at the 1% significance level. That is, ownership of PHI is an endogenous variable, which required unbiased estimation using IV. Next, the correlation between the IV and the endogenous explanatory variables was determined. The *F* value of the first stage regression was 69.25, which was much larger than the conventional critical value of 10. This outcome implied that there was no weak IV and that the IV we chose had strong explanatory power for the endogenous variables. In addition, given that there was only one IV, it was exactly identified.

The results of the second stage regression showed that after introducing the IV to adjust for the estimation bias, participation in PHI still had a positive effect on the health status of the population and the return grew to 8.21% (Table 4). The influence coefficient estimated by FE model with IV was more than seven times that of the FE model, indicating that the FE model had a large deviation. The traditional panel regression analysis ignored the possible endogenous problems, thus weakening the health promotion effect of participating in PHI on residents.

**Table 4:** The FE model with IV results of the effect of having PHI.

**Variables**	**FE**+**IV**	
	**The first stage**	**The second stage**
The ownership of PHI	—	0.0821***(0.0102)
Gender	—	—
Age	−0.0024 (0.0045)	−0.0400(0.0034)
Marital status	0.0051 (0.0040)	0.0035*** (0.0030)
Education	−0.0041 (0.0046)	0.0022 (0.0035)
Registered permanent residence	−0.0008 (0.0042)	−0.0105*** (0.0032)
Smoke	−0.0103 (0.0072)	−0.0305*** (0.0055)
Drink	0.0049* (0.0030)	0.0429*** (0.0022)
Social contact	−0.0016 (0.0028)	0.0446*** (0.0022)
Disease	0.0044 (0.0028)	−0.0606*** (0.0021)
Household income per capita	0.0013 *** (0.0004)	0.0004 (0.0003)
IV	1.0793*** (0.0146)	—
*F* test: *F*	83.07***	
Hausman test: *χ^2^*	69.25***	
Observations	44,397	

### Results of robustness test by changing the dependent variable

The test results of the IV showed that “participation rate of PHI in the community” passed the endogeneity test (χ^2^ = 80.42, *p* < 0.001) and the weak IV test (*F* = 45.42, *p* < 0.001). The results of robustness analysis showed that participating in PHI could significantly improve residents' self-assessed health status by 60%, which was basically consistent with the previous conclusion with “EQ-5D index scores” as the explained variable (Table 5). The result was relatively robust.

**Table 5:** The effects of having PHI on the self-assessed health of residents.

**Variables**	**FE**+**IV**	
	**The first stage**	**The second stage**
The ownership of PHI	—	0.6006 (0.0879)
Gender	—	—
Age	−0.0059 (0.0066)	0.6438 (0.0284)
Marital Status	0.0085 (0.0060)	−0.0119 (0.0257)
Education	0.0000 (0.0066)	0.0242 (0.0282)
Registered permanent residence	−0.0013 (0.0058)	0.0195 (0.0250)
Smoke	−0.0100 (0.0111)	0.5543 (0.0477)
Drink	−0.0001 (0.0045)	0.1894 (0.0192)
Social contact	−0.0062 (0.0043)	0.0781 (0.0183)
Disease	−0.0015 (0.0042)	−0.6226 (0.0181)
Household income per capita	0.0008 (0.0007)	0.0503 (0.0029)
IV	1.0870*** (0.0223)	—
*F* test: *F*	80.42***	
Hausman test: *χ^2^*	45.42 ***	
Observations	23,726	

### Heterogeneity analysis of the effects of PHI on different populations

Considering that chronic disease might affect the health utility gain of PHI, the samples were divided into those with and without chronic diseases, and the FE model with IV was used for regression analysis. The results showed that, consistent with the full-sample analysis, participating in PHI had a significant positive effect on both sub-samples. However, the health gain of participating in PHI was 9.25% for the sample group with chronic disease and 6.24% for the non-chronic disease group, indicating that the effect of participating in PHI on residents' health had the heterogeneity on the group with and without chronic diseases, especially for the group with chronic disease (Table 6).

**Table 6:** The effects of PHI on health status of residents with and without chronic diseases.

**Variables**	**With chronic** **diseases**	**Without chronic** **diseases**
The ownership of PHI	0.0925*** (0.0182)	0.0624*** (0.0179)
Gender	—	—
Age	−0.0399*** (0.0062)	−0.0411*** (0.0058)
Marital status	0.0020 (0.0054)	−0.0026 (0.0053)
Education	0.0022 (0.0065)	0.0060*** (0.0059)
Registered permanent residence	−0.0082 (0.0061)	−0.0158*** (0.0053)
Smoke	−0.0356*** (0.0104)	−0.0301*** (0.0091)
Drink	0.0560*** (0.0041)	0.0250*** (0.0038)
Social contact	0.0503*** (0.0038)	0.0342*** (0.0038)
Household income per capita	0.0004 (0.0006)	0.0009 (0.0006)
IV	Yes	Yes

We also assessed the effect of PHI on the insured at different income levels. The median annual household income per capita of all samples was calculated. Samples with annual household income per capita equal to or above the median were classified as the high-income group, and samples with annual household income per capita below the median were classified as the low-income group. The results of sub-sample regression showed that owning PHI had a significant effect on individual health in different income groups. However, such an effect was significantly different between low- and high-income income groups. PHI improved the EQ-5D index scores of the high-income group by 8.32%; low-income group, 5.31%. The health effect of PHI was particularly obvious in high-income groups with better economic status, and relatively small in low-income groups (Table 7).

**Table 7:** The effects of PHI on health status of low- and high-income residents.

**Variables**	**Low income**	**High income**
The ownership of PHI	0.0531*** (0. 0185)	0.0832*** (0.0178)
Gender	—	—
Age	−0.0369*** (0.0067)	−0.0470*** (0.0058)
Marital status	0.0074 (0.0056)	0.0022 (0.0055)
Education	0.0074 (0.0077)	0.0016 (0.0059)
Registered permanent residence	−0.0182* (0.0071)	−0.0116** (0.0052)
Smoke	−0.0316*** (0.0104)	−0.0249*** (0.0095)
Drink	0.0435*** (0.0041)	0.0472*** (0.0038)
Social contact	0.0430*** (0.0040)	−0.0479*** (0.0039)
Disease	−0.0653*** (0.0040)	−0.0585*** (0.0039)
IV	Yes	Yes

## Discussion

Based on the “choice hypothesis”, healthier people are more likely to purchase supplemental PHI, so the role of PHI for health promotion is controversial (32, 33). Using the quasi-experimental method, we excluded the endogenous interference of insurance adverse selection. We found that PHI could still significantly improve the comprehensive health status of residents, which verified our first hypothesis. This is because benefits of PHI tend to be better than SHI (34), with higher cost sharing and a wider range of services, thus influencing health through the mediating effect of health-care service utilization. Two possible paths of action exist for this mediation. Some scholars believe that PHI has fewer treatment thresholds than SHI, and PHI covers medical expenses not included in SHI, allowing the insured to actively accept outpatient and inpatient treatment services when they experience health problems (35–37). However, many studies showed that the insured tends to utilize services that are exclusively covered by PHI, such as physical examinations, cancer screening, innovative drugs, and so on (38, 39), to better manage their current health conditions. The results revealed that although the use of relatively expensive services such as hospitalization did not increase (40), the insured received more valuable health services and reduced financial risks (41, 42). Contrastingly, the second path is more cost effective, consistent with the current popular and advanced concept of value-based insurance design (VBID) (30). VBID promotes the utilization of high-value preventive services by reducing or eliminating cost sharing for the insured for high-value services and increasing out-of-pocket costs for low-value services, resulting in higher health benefits at less cost (43, 44). Currently, domestic scholars are paying less attention to the coverage of preventive health care services of PHI and the incentives for patients to use such services, and pay more attention to medical services. It is necessary for scholars to change their research perspective. The government should strengthen the guidance of commercial insurance companies to explore VBID, which is highly significant to the cost control of insurance companies, the health of patients, and the resource saving of the health system.

We also found that participation in PHI had greater health gains for people with chronic diseases, verifying hypothesis 2 as well. Chronic diseases are associated with multiple health risks such as poor functional status, poor quality of life and increased psychological distress (45, 46). Chronic patients often suffer huge economic burden accompanied by continuous expenditure of long-term health service needs (41, 47). The economic pressure may force patients to reduce other expenditures in their lives to pay for health care or delay their visits, thus further endangering their health, which is unsolvable by SHI (48). Compared with the general population, health insurance plays a more significant role in dispersing the risk of medical expenses for patients with chronic diseases. As discussed above, PHI can increase the access of patients with chronic diseases to diagnostic and preventive services before complications occur (49), preventing greater damage to their health. At present, about 180 million elderly people in China suffer from chronic diseases (50), and deaths caused by chronic diseases have accounted for 86.6% of the total number of deaths in the country (51). Moreover, the total economic burden of chronic diseases is on the rise, and its growth rate has outpaced the growth rate of GDP. The rapid growth of health costs and the risk of life and health caused by chronic diseases have become an important issue of high concern to all countries. The role of PHI may be conducive to alleviating the threat of chronic diseases. However, PHI suffers from market failure, as evidenced by its “skimming” behavior. Commercial insurance companies that are profit drive, would cherry pick the young and healthy and exclude or impose onerous conditions on the elderly and infirm (52). The government ought to carry out strict supervision to prohibit commercial insurance companies from selectively selling insurance products by means of the so-called “actuarial calculation” on the basis of consumers' personal information such as health status, resulting in the exclusion of patients with chronic and serious illnesses who really need PHI and undermining the risk diversification mechanism of health insurance.

Finally, our hypothesis 3 was also verified that PHI provided less health gain to low-income groups than to high-income groups. This difference is because low-income groups, limited by their ability to pay, tend to choose PHI with lower premiums and lower levels of protection. Buchmueller also reported that consumers who purchased PHI are much more likely to purchase other types of insurance (53). Even if the medical expenses are shared by PHI, low-income groups still worry about their out-of-pocket expenses. Lee et al. pointed out that low-income families have more unmet medical service experience (54). Higher incomes increase the likelihood of seeking more health care and paying for it. In addition, low-income groups face more health threats such as increased psychological distress, less social support, higher intensity of work, and less healthy diets (55, 56), which may lead to less significant health gains from supplementary PHI. For China, the low-income population, especially the rural population, is relatively large and faces huge risks. The security of the low-income group is the core of the social security system and plays a positive role in promoting economic development and social stability (57). Based on the national conditions, PHI providers should prioritize the protection of low-income people and promote the inclusive innovation of PHI, especially the inclusive products aimed at low-income groups. In addition, PHI providers should lower the premium threshold for low-income people to expand the coverage and level of insurance.

## Advantages and limitations

On the basis of eliminating endogeneity, we analyzed the value of PHI in improving residents' health and explored the heterogeneity of this effect in different groups of populations. This study provides supportive evidence for the improvement of PHI policy and the development of PHI in China and encourages the public to actively participate in PHI.

The study also had several limitations. Firstly, the data we used came from the public database of the survey conducted by Peking University rather than our own survey. Due to data limitations and other factors, we only selected an IV, and some key control variables, such as the classification of SHI and the utilization of outpatient or inpatient visits, were not included. Secondly, we only discussed the results of the effect of the insurance on health. The mediating effect and other influencing mechanisms were not studied. These issues will be considered in future studies. Thirdly, although we used composite health indicators as dependent variables to minimize self-reported bias, it was still impossible to completely avoid bias.

## Conclusion

After excluding the possible two-way causal relationship between insurance and health status, we found that PHI still promoted health status and had a more significant effect on patients with chronic diseases. This finding proves the health value of PHI. However, PHI can bring health inequality to different income groups. Therefore, PHI should be developed on the basis of SHI. The government can support the development of PHI by providing insurance incentives for individuals who buy PHI and doing a good job in connecting SHI and PHI systems. In addition, PHI providers are encouraged to actively develop health insurance products exclusively for the elderly to meet the insurance needs of senior citizens. Moreover, the threshold for low-income people to participate in PHI should be lowered to expand the scope and level of insurance.

## Data availability statement

Publicly available datasets were analyzed in this study. This data can be found at: http://charls.pku.edu.cn.

## Author contributions

XinlinC, DG, HT, and YC conceived and designed the study, YZ, YL, and XinlanC cleared up and analyzed the data. XinlinC, YC and DG wrote the first draft. All authors supplied critical revisions to the manuscript and gave final approval of the version to be published.

## Funding

This study was supported by National Natural Science Foundation of China (Support batch number: 71974066) and Double First-class Construction Project of Liberal Arts in Huazhong University of Science and Technology (Think Tank of Rural Health Service Policy and Management).

## Conflict of interest

The authors declare that the research was conducted in the absence of any commercial or financial relationships that could be construed as a potential conflict of interest.

## Publisher's note

All claims expressed in this article are solely those of the authors and do not necessarily represent those of their affiliated organizations, or those of the publisher, the editors and the reviewers. Any product that may be evaluated in this article, or claim that may be made by its manufacturer, is not guaranteed or endorsed by the publisher.

## Nomenclature

Countries around the world are trying to improve their health care systems. Some countries such as China, France and Belgium are developing a system with social health insurance as the main body and private health insurance as the supplement. We explored the effect of private health insurance on residents' health, which is conducive to providing scientific basis for the formulation of policies related to private health insurance. Secondly, it is controversial whether private health insurance can improve the health of the insured. Our study confirmed that participating in private health insurance on the basis of social health insurance can indeed further improve the health level of the insured, and provide greater health gain for the chronic disease population, which provides guidance and incentive for residents to participate in insurance. In addition, we explore the differences in the impact of private health insurance on the health of different populations with a view to providing evidence for promoting health equity.
