# Supplementary material for: Can supplementary private health insurance further supplement health
Source: Front Public Health. 2022 Sep 27;10:961019. doi: 10.3389/fpubh.2022.961019 (PMC9552012; doi:10.3389/fpubh.2022.961019)
Supplement: Supplementary file 1 [file Table_1.DOCX]

Supplementary Material

**Supplementary Table 1**

Table 1 Definition/codes of the control variables

| Dimension | Variables | Definition |
| --- | --- | --- |
| Socioeconomic characteristics | Gender | 0=Female; 1=Male |
|  | Age | 1=The young (18-44 years); 2= The middle-aged(45-59 years); 3=Younger elderly (60-74 years); 4=The elderly(≥75 years) |
|  | Marital Status | 0 = Unmarried; 1 = Married |
|  | Education | 1 = Junior high school and below; 2 = Vocational/Technical secondary school/High school; 3 = Junior college and above |
|  | Registered permanent residence | 0 = Non-rural household registration;  1 = rural household registration |
|  | household incomes per capita | Continuous variable of the logarithm of annual per capita income of household members |
| Health-related behavior | Smoke | 0 = No; 1 = Yes |
|  | Drink | 0 = No; 1 = Yes |
|  | Social contact | 0 = No; 1 = Yes |
| Disease status | Chronic disease | 0 = No; 1 = Yes |
